# The impact of face shields on the quality of gastrointestinal endoscopy during the COVID-19 pandemic

**DOI:** 10.1186/s12876-022-02114-2

**Published:** 2022-01-29

**Authors:** Jong Yoon Lee, Yeo Wool Kang, Myeongseok Koh, Dong Kyun Kim, Jin Seok Jang, Jong Hoon Lee

**Affiliations:** Department of Internal Medicine, Dong-A University College of Medicine, Busan, 49201 South Korea

**Keywords:** Adenomatous polyps, Colonic polyps, Colonoscopy, COVID-19, Endoscopy

## Abstract

**Background:**

Coronavirus disease 2019 (COVID-19) has become a global pandemic, with healthcare workers at a high risk of exposure. During this pandemic, endoscopists must wear personal protective equipment (PPE), including face shields, to prevent COVID-19 transmission; however, few studies have reported the impact of face shields on the quality of gastrointestinal (GI) endoscopy. We aimed to determine whether the use of PPE, including face shields, affected the quality of GI endoscopy during the COVID-19 pandemic.

**Methods:**

The medical records of patients who had undergone screening or surveillance colonoscopy and gastric endoscopic submucosal dissection (ESD) at Dong-A University Hospital between June 2020 and March 2021 were retrospectively reviewed. Endoscopists wore isolation gowns, disposable gloves, and KF94 masks from June 2020 to October 2020. From November 2020, endoscopists also wore face shields. We compared GI endoscopy quality indicators between the first five months (no face shields) and the second five months (with face shields). In the non-face shield and face shield groups, we calculated the overall adenoma detection rates (ADRs), polyp detection rate (PDR), sessile serrated lesion detection rate (SSLDR), advanced neoplasia detection rate (ANDR), complete resection rate (CRR), number of polyps and/or adenomas per colonoscopy, and gastric ESD procedure time.

**Results:**

In total, 1359 study patients had undergone screening or surveillance colonoscopy (face shield group, n = 679; non-face shield group, n = 680). No statistically significant between-group differences were observed (PDR, 49.04 vs. 52.50%, *p* = 0.202; ADR, 38.59 vs. 38.97%, *p* = 0.884; SSPDR, 1.91 vs. 1.32%, *p* = 0.388; ANDR, 3.98 vs. 3.97%, *p* = 0.991, respectively). No difference was found in colonoscopy quality indicators between patients examined by experienced and trainee endoscopists with and without face shields. Of 144 study patients who had undergone gastric ESD for gastric neoplasms, there were 72 patients in each group. No statistically significant differences were found in the CRR (94.44 vs 93.05%, *p* = 1.000) and procedure times (19.22 ± 9.33 vs. 19.03 ± 11.49, *p* = 0.911).

**Conclusions:**

Wearing face shields during the COVID-19 pandemic did not affect the quality indicators for GI endoscopy.

## Background

In December 2019, an outbreak of severe acute respiratory syndrome coronavirus 2 (SARS-CoV-2) was detected in a cluster of patients with respiratory tract infection of unknown etiology in Wuhan, Hubei Province, China [1]. In February 2020, the World Health Organization named the infection due to SARS-CoV-2 as coronavirus disease 2019 (COVID-19) and declared COVID-19 to be a pandemic in March 2020 [2]. COVID-19 is mainly transmitted through droplets, aerosols, and via direct contact; therefore, healthcare workers are at a higher risk of exposure to COVID-19 than the general population [3, 4]. Fecal-to-oral transmission may also be a route of COVID-19 infection [5]; therefore, endoscopic procedures increase the risk of COVID-19 transmission. The World Endoscopy Organization, the American Society for Gastrointestinal Endoscopy, the American Gastroenterological Association, the European Society of Gastrointestinal Endoscopy, and the Asian Pacific Society for Digestive Endoscopy have made several recommendations for clinicians to follow during the COVID-19 pandemic that include assessing the risk of COVID-19 to determine when to perform an endoscopy and recommending that all endoscopists wear appropriate personal protective equipment (PPE) to prevent COVID-19 transmission [6–10]. PPE includes an isolation gown, disposable gloves, a mask, and a face shield or goggles. A face shield is used for protection of the facial area from exposure to infectious agents. Many protective devices had previously been used during endoscopy, but face shields had not been used prior to the COVID-19 pandemic.

Colonoscopy is the gold standard method for the screening and diagnosis of colorectal cancer (CRC) [11, 12]. However, poor quality colonoscopy can lead to post-colonoscopy CRC (PCCRC) [13]. An increased adenoma detection rate (ADR) has been reported to reduce the risk of PCCRC and CRC-related mortality [14, 15]. The resolution of the colonoscope is known to affect the ADR [16, 17]. High resolution provides clear images, making it easy to detect abnormalities in the colonic mucosa. The sharpness of the screen is an important factor influencing the ADR during colonoscopy [18]. Since the face shield is a device worn in front of the eyes, the visual field of the user observing the screen may be affected. Compared with colonoscopy, there are few useful indicators to evaluate the quality of upper gastrointestinal (GI) endoscopy. We evaluated the complete resection rate (CRR) in gastric endoscopic submucosal dissection (ESD) as a quality indicator in upper GI endoscopy. ESD is a widely accepted treatment for early gastric cancer or gastric adenoma. Accurate delineation of margins is necessary for complete resection of gastric neoplasms [19, 20]. However, this requires precise observation, and there has been concern that wearing a face shield may affect accurate delineation. Few studies have reported the effect of face shields on the quality of GI endoscopy; therefore, we aimed to determine whether the use of a face shield affected GI endoscopy quality.

## Methods

### Study design and patients

We retrospectively reviewed the medical records of patients who had undergone screening or surveillance colonoscopy and gastric ESD at Dong-A University Hospital between June 2020 and March 2021 during the COVID-19 pandemic. All patients who had undergone colonoscopy were aged 30–79 years and had undergone either their first screening colonoscopy or surveillance colonoscopy after three years of their last examination. Colonoscopies were performed by four experienced endoscopists with > 5 years of experience and by three second- or third-year gastroenterology fellows who were able to perform colonoscopies independently. Patients aged ≥ 30 years who had undergone gastric ESD for the treatment of category 4 (mucosal high grade dysplasia or intramucosal carcinoma) disease according to the revised Vienna classification and confirmed after gastric ESD were included [21]. Gastric ESD was performed by two experienced endoscopists.

**Fig. 1:**
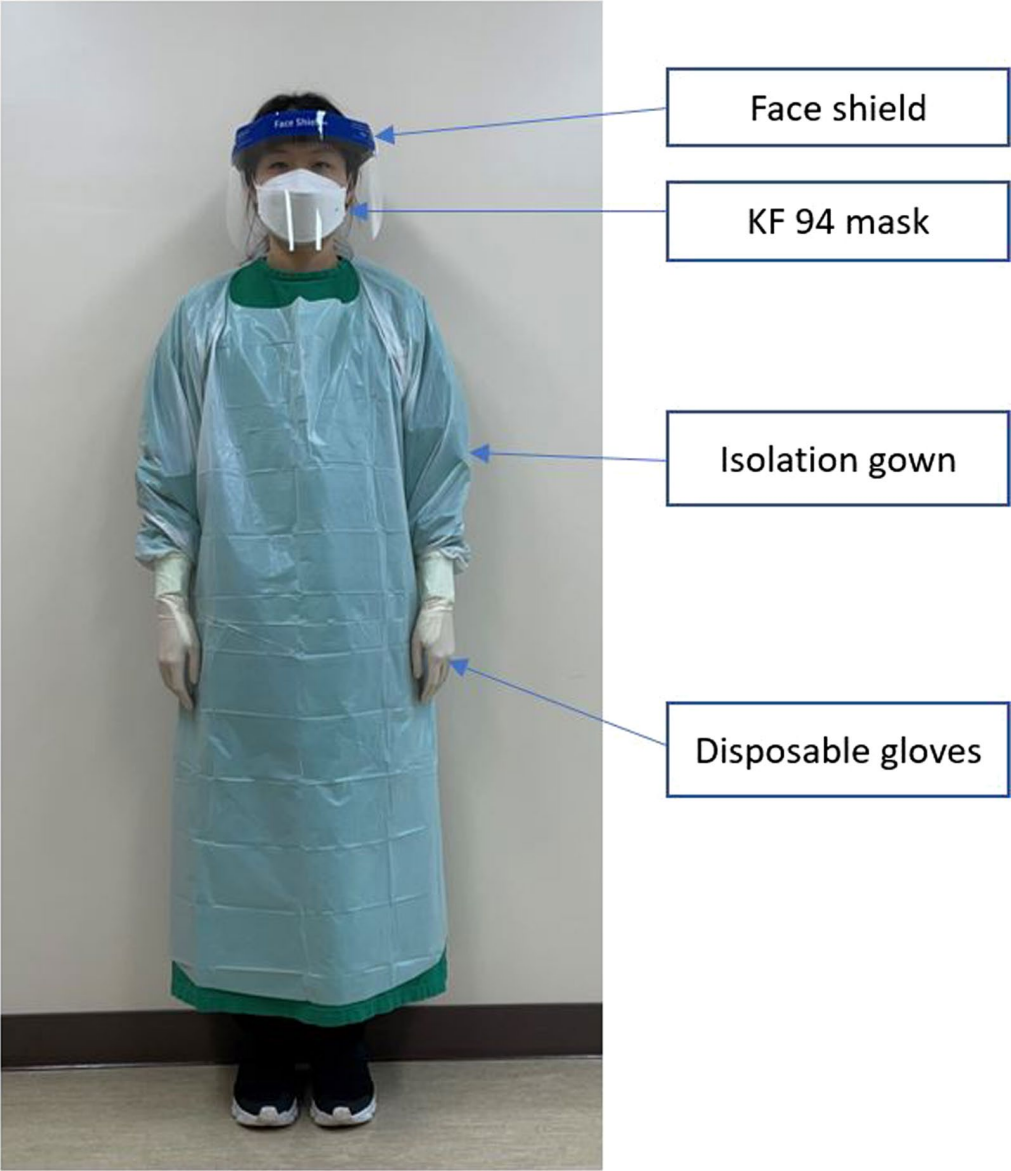
Endoscopists wore isolation gowns, disposable gloves, and KF94 masks from June 2020 to October 2020. From November 2020, endoscopists additionally wore face shields

The endoscopists wore isolation gowns, disposable gloves, and KF94 masks from June 2020 to October 2020; patients examined under this condition were classified into a non-face shield group. From November 2020 onwards, the endoscopists additionally wore face shields, and the patients examined under this condition were classified into a face shield group (Fig. 1). We compared the quality indicators of GI endoscopy during five months without the use of face shields and five months with the use of face shields.

### Risk stratification and endoscopic procedures

Dong-A University Hospital is a tertiary hospital that treats patients with COVID-19. Since the start of the COVID-19 pandemic, our hospital has classified patients’ risk of COVID-19 infection into three categories. Low-risk patients comprise those with no symptoms (e.g., cough, temperature > 37.5°, breathlessness, diarrhea), no contact with COVID-19-positive patients, and not having stayed in high-risk areas in the previous 14 days. Intermediate-risk patients comprise those with symptoms, but with no contact with COVID-19-positive patients, and not having stayed in high-risk areas in the previous 14 days, or patients without symptoms but who had contact with COVID-19-positive patients or who had stayed in high-risk areas in the previous 14 days. High-risk patients comprise those with symptoms and who had contact with COVID-19-positive patients or who had stayed in high-risk areas in the previous 14 days. During the chart review, colonoscopy and gastric ESD were performed for low-risk patients. Intermediate- or high-risk patients underwent a polymerase chain reaction test for COVID-19 to confirm a negative test result, and the necessity of endoscopy was evaluated. Subsequently, endoscopy was performed for patients with negative results but not for those confirmed as being positive for COVID-19. High-definition video processor systems (i.e., CV-290 EVIS LUCERTA ELITE [Olympus Medical, Tokyo, Japan] and EPK-i7010 [Pentax, Hoya Corporation, Tokyo, Japan]) were used for all coloscopies. However, when gastric ESD was performed, only the CV-290 EVIS LUCERTA ELITE [Olympus Medical, Tokyo, Japan] was used because routine observation using a narrow band image (NBI) was required. Patients undergoing colonoscopy were prepared with 1–2 L of a polyethylene glycol solution containing ascorbic acid with an additional 1–2 L of water. Midazolam 2–5 mg and/or propofol 10–60 mg was administered for sedation purposes.

### Definitions concerning the polyp detection rate, the ADR, the sessile serrated lesion detection rate, and the advanced neoplasia detection rate

The polyp detection rate (PDR) was defined as the proportion of patients with at least one polyp, including adenoma and hyperplastic polyps (HPs), among all the patients examined. The ADR was defined as the proportion of patients with at least one adenoma among all the patients examined. We added features of clinically significant sessile serrated lesions (SSLs) to our definition of SSL. Therefore, the SSL was defined as follows: (a) a SSL with or without dysplasia, (b) an HP measuring ≥ 5 mm in the proximal colon (proximal to the splenic flexure), or (c) an HP ≥ 10 mm in the whole colon. Advanced adenoma was defined as follows: any adenoma ≥ 10 mm in size, with villous histology or with high-grade dysplasia, and any SSL ≥ 10 mm in size or with dysplasia. The SSL detection rate (SSLDR) and the advanced neoplasia detection rate (ANDR) were calculated in a similar manner to the PDR and the ADR.

### Evaluation of horizontal margins and definition of the CRR

The CRR was calculated to evaluate whether the horizontal extent of the gastric neoplasm was adequately observed when performing ESD as the quality indicator that could most affect the visual field in upper GI endoscopy. Prior to performing an ESD, the horizontal margins were evaluated using two methods, namely, a white light image and an NBI. A pathological analysis of the resected specimen was defined as a complete resection that included the horizontal margins of the neoplasm.

### Primary and secondary endpoints

The primary endpoints were the comparison of the ADR and the CRR between the groups. The secondary endpoints were the PDR, SSLDR, ANDR, number of adenoma per colonoscopy (APC), intubation time, and withdrawal time in lower GI endoscopy. A comparison of the procedure time for gastric ESD was also a secondary endpoint.

### Statistical and data analyses

The data were divided into two groups: data from procedures performed wearing face shields and data from procedures performed without face shields. Continuous data were analyzed using a Student’s *t*-test and are represented as mean ± standard deviation. Categorical data were analyzed using Pearson’s chi-squared or Fisher’s exact tests. All analyses were performed using the Statistical Package for Social Sciences software (version 26.0, IBM, Armonk, New York, USA). Statistical significance was set at *p* < 0.05.

### Ethics statement

Our research protocol was approved by the Ethics Committee of our hospital in accordance with international agreements (World Medical Association Declaration of Helsinki: Ethical principles for medical research involving human subjects). Due to the retrospective characteristics of the study, informed consent was waived, and the study was approved by the Institutional Review Board of Dong-A University College of Medicine (DAUHIRB-21-110).

## Results

### Colonoscopy quality indicators between the groups

In total, 1359 patients were included in this study; 680 and 679 procedures were performed without and with face shields, respectively. The demographic and clinical characteristics of the patients are summarized in Table 1. There were no significant differences between the two groups (face shield vs. non-face shield) regarding patients’ age and sex, bowel preparation, sedation, use of antiplatelet or anticoagulation, time of examination, indication for examination, type of colonoscope, and endoscopist’s experience (Table 1). There were no significant differences in insertion time between the face shield and non-face shield groups (431.94 ± 199.08 vs. 431.71 ± 209.18, respectively; *p* = 0.983) and withdrawal time (524.97 ± 164.55 vs. 537.54 ± 164.20, respectively; *p* = 0.159). There were no significant differences between the face shield and non-face shield groups in terms of the PDR (49.04 vs. 52.50%, respectively; *p* = 0.202), ADR (38.59 vs. 38.97%, respectively; *p* = 0.884), SSLDR (1.91 vs. 1.32%, respectively; *p* = 0.388), ANDR (3.98 vs. 3.97%, respectively; *p* = 0.991), number of polyps per colonoscopy (1.11 ± 1.83 vs. 1.10 ± 1.57*,* respectively; *p* = 0.897), and number of APC (0.73 ± 1.34 vs. 0.69 ± 1.12, respectively; *p* = 0.471) (Table 2).

**Table 1:** Demographic and clinical characteristics in patients who underwent colonoscopy

	Face shield (n = 679)	Non-face shield (n = 680)	*P* value
Age	61.56 ± 11.32	62.68 ± 11.07	0.067
Gender			0.803
Male	381	377	
Female	298	303	
Bowel preparation			0.565
BBPS 8,9	377	367	
BBPS 6,7	302	313	
Sedation			0.705
Yes	666	665	
No	13	15	
Antiplatelet or anticoagulation use			0.937
Yes	71	72	
No	608	608	
Examination of day			0.487
Morning	183	172	
Afternoon	496	508	
Reason of examination			0.461
Screening	516	505	
Surveillance	163	175	
Type of colonocope			0.846
CV-290	300	304	
EPK-i7010	379	376	
Endoscopist’s experience			0.683
Experienced	317	325	
Trainee	362	355	

*BPPS* Boston bowel preparation score

**Table 2:** Colonoscopic quality indicators between the groups

	Face shield (n = 679)	Non-face shield (n = 680)	*P* value
Cecal intubation time (second)	431.94 ± 199.08	431.71 ± 209.18	0.983
Withdrawal time (second)	524.97 ± 164.55	537.54 ± 164.20	0.159
PDR (%)	49.04 (333/679)	52.50 (357/680)	0.202
ADR (%)	38.59 (262/679)	38.97 (265/680)	0.884
SSLDR (%)	1.91 (13/679)	1.32 (9/680)	0.388
ANDR (%)	3.98 (27/679)	3.97 (27/680)	0.991
PPC	1.11 ± 1.83	1.10 ± 1.57	0.159
APC	0.73 ± 1.34	0.69 ± 1.12	0.471
PPB, %	0/333	0/357	1.000

*PDR* polyp detection rate, *ADR* adenoma detection rate, *SSPDR* sessile serrated polyp detection rate, *ANDR* advanced neoplasm detection rate, *PPC* polyp per colonoscopy, *APC* adenoma per colonoscopy, *PPB* post polypectomy bleeding

### Adenomas per colonoscopy according to endoscopic features and size

Adenomas were divided into two categories according to their endoscopic features: polypoid and flat. The number of APC, according to endoscopic features, was not significant between the face shield and non-face shield groups (polypoid: 0.05 vs. 0.05, respectively; *p* = 0.933; flat: 0.68 vs. 0.64, respectively; *p* = 0.457). Adenomas were divided into three categories according to size: < 5 mm, 5–10 mm, and > 10 mm. The number of APC, according to size, did not differ significantly between the face shield and non-face shield groups (< 5 mm: 0.66 vs. 0.58, respectively; *p* = 0.199; 5–10 mm: 0.05 vs. 0.08, respectively; *p* = 0.171; > 10 mm: 0.03 vs. 0.03, respectively, *p* = 0.997; Table 3).

**Table 3:** Adenomas per colonoscopy by endoscopic feature and size

	Face shield (n = 679)		Non-face shield (n = 680)	*P* value
Endoscopic feature				
Polypoid		0.05 (31/679)	0.05 (34/680)	0.933
Flat		0.68 (465/679)	0.64 (435/680)	0.457
Size				
< 5 mm		0.66 (445/679)	0.58 (394/680)	0.199
5– 10 mm		0.05 (31/679)	0.08 (54/680)	0.171
> 10 mm		0.03 (20/679)	0.03 (21/680)	0.997
Overall		0.73 (496/679)	0.69 ± 1.12	0.471

*APC* adenoma per colonoscopy

### Colonoscopy quality indicators according to endoscopists’ experience

Data concerning the face shield and non-face shield groups were further compared according to endoscopists’ experience. Regarding trainee endoscopists, there were no significant differences between the face shield and non-face shield groups in cecal intubation time (455.02 ± 199.81 vs. 464.22 ± 185.30, respectively; *p* = 0.523), withdrawal time (522.68 ± 172.04 vs. 544.33 ± 172.97, respectively; *p* = 0.093), the PDR (46.69 vs. 51.55, respectively; *p* = 0.193), the ADR (35.36 vs. 37.46, respectively; *p* = 0.558), and the number of APC (0.61 ± 1.23 vs. 0.63 ± 1.10, respectively; *p* = 0.789). Similarly, regarding experienced endoscopists, there were no statistically significant differences between the face shield and non-face shield groups in cecal intubation time (405.58 ± 195.25 vs. 396.19 ± 227.50, respectively; *p* = 0.575), withdrawal time (527.58 ± 155.78 vs. 530.13 ± 153.98, respectively; *p* = 0.835), the PDR (52.29 vs. 53.53, respectively; *p* = 0.647), the ADR (42.22 vs. 40.61, respectively; *p* = 0.670), and the number of APC (0.88 ± 1.44 vs. 0.74 ± 1.14, respectively; *p* = 0.198; Table 4).

**Table 4:** Colonoscopic quality indicators according to endoscopists’ experience

	Face shield	Non-face shield	*P* value
Trainee endoscopts			
Cecal intubation time, s	455.02 ± 199.81	464.22 ± 185.30	0.523
Withdrawal time, s	522.68 ± 172.04	544.33 ± 172.97	0.093
PDR, %	46.69	51.55	0.193
ADR, %	35.36	37.46	0.558
APC	0.61 ± 1.23	0.63 ± 1.10	0.789
Experienced endoscopists			
Cecal intubation time, s	405.58 ± 195.25	396.19 ± 227.50	0.575
Withdrawal time, s	527.58 ± 155.78	530.13 ± 153.98	0.835
PDR, %	52.29	53.53	0.647
ADR, %	42.22	40.61	0.670
APC	0.88 ± 1.44	0.74 ± 1.14	0.198

*PDR* polyp detection rate, *ADR* adenoma detection rate, *APC* adenoma per colonoscopy

### The CRR and the procedure time of gastric ESD between the groups

Of 144 patients who underwent gastric ESD for gastric neoplasms in this study, 72 patients were in the face shield group and 72 patients were in the non-face shield group. No significant difference was observed in terms of demographics and clinical characteristics between the two groups (Table 5). No statistically significant differences were found between the two groups in the CRR (94.4 vs 93.05%, respectively, *p* = 1.000) and procedure time (19.22 ± 9.33 vs. 19.03 ± 11.49, respectively, *p* = 0.911; Table 6).

**Table 5:** Demographics and clinical characteristics in patient who underwent gastric ESD

	Face shield (n = 72)	Non-face shield (n = 72)	*P* value
Age, mean, years	65.67 ± 9.30	67.35 ± 10.18	0.303
Gender, n			0.835
Male	58	57	
Female	14	15	
Tumor size, mean ± SD, mm	12.70 ± 7.21	14.89 ± 8.43	0.097
Location			0.067
Antrum	46	56	
Body and fundus	26	16	
Macroscopic type			0.943
Elevated	30	32	
Flat	12	9	
Depressed	21	24	
Mixed	9	7	
H.pylori status			0.863
Positive	26	27	
Negative	46	45	
Final pathology			0.165
HGD	21	12	
EGC, differentiated	48	58	
EGC, undifferentiated	3	2	
Invasion depth			0.574
HGD and T1a	66	64	
T1b and deeper	6	8	
Endoscopists			0.688
Endoscopist 1	57	55	
Endoscopist 2	15	17	

*EGC* early gastric cancer, *HGD* high grade dysplasia

**Table 6:** Procedure time and complete resection rate between groups

	Face shield	Non-face shield	*P* value
Endoscopist 1			
Procedure time, min	18.65 ± 9.44	18.05 ± 10.24	0.750
Complete resection rate, %	92.98	94.55	1.000
Negative lateral margin	53	52	
Positive lateral margin	4	3	
Endoscopist 2			
Procedure time, min	21.40 ± 8.88	22.18 ± 14.77	0.861
Complete resection rate, %	100	88.24	0.486
Negative lateral margin	15	15	
Positive lateral margin	0	2	
Overall			
Procedure time, min	19.22 ± 9.33	19.03 ± 11.49	0.911
Complete resection rate, %	94.44	93.05	1.000
Negative lateral margin	68	67	
Positive lateral margin	4	5	

## Discussion

This single-center retrospective study aimed to determine whether the use of a face shield affected the quality of GI endoscopy during the COVID-19 pandemic. We found that performing endoscopic procedures while wearing a face shield did not affect the quality indicators of GI endoscopy. In addition, the proficiency of both experienced endoscopists and trainee endoscopists was not affected by the use of face shields.

As the number of endoscopic procedures performed increases, occupation-associated health hazards for endoscopists have increased, one of which is exposure to infection [22]. Throughout the COVID-19 pandemic, endoscopists have been at an increased risk of contracting COVID-19 from airborne droplets and conjunctival contact. Because human-to-human transmission occurs primarily through direct contact or air droplets, upper GI endoscopy may be a procedure that increases the risk of COVID-19 infection due to patients coughing during the examination [7, 23]. The live SARS-CoV-2 virus has also been found in patient stools, and fecal–oral transmission of COVID-19 is also possible [5, 24]. Therefore, colonoscopy is likely to be a procedure involving an increased risk of COVID-19 infection. Furthermore, patients with COVID-19 can present clinically with atypical GI symptoms; therefore, endoscopy may be performed on an undiagnosed, infected patient with COVID-19, thereby further increasing the risk of COVID-19 transmission [25].

One study that quantified the rate of unrecognized exposure to potentially infectious biologic samples during endoscopy via an endoscopist’s face reported that facial exposure may result in transmission of infectious diseases [26]. According to previous studies conducted in the early phase of the COVID-19 pandemic, 19% of healthcare workers who wore masks and gloves and who performed hand hygiene without additional facial protection were infected with COVID-19, but those who used additional facial protection were not infected [27, 28]. For these reasons, it is important that endoscopists wear a face shield along with isolation gowns, gloves, and a mask throughout endoscopic procedures during this COVID-19 pandemic.

However, wearing a face shield may affect the observation capacity of endoscopists during procedures. Previous studies have reported that the ADR was affected by the resolution and visual field of the colonoscopy [16–18]. Observing adenomas is relatively uncomplicated when a screen is clear and the visual field is wide. However, concerns may be raised regarding whether the wearing of a face shield affects clarity or the visual field during colonoscopy and thus reduces the ADR. Therefore, we aimed to determine whether wearing a face shield affected endoscopists’ performance and whether the procedure time subsequently increased or whether the withdrawal time subsequently decreased. During our study period, Lee et al. reported that colonoscopy performance was not unfavorably affected when wearing a face shield [29]. We also found that wearing a face shield did not affect the quality indicators of colonoscopy, including the ADR. However, our study differed from Lee et al.’s study in that we analyzed quality indicators concerning both lower and upper GI endoscopy. Endoscopic retrograde cholangiography was excluded from our analysis because wearing goggles had been recommended prior to the COVID-19 pandemic due to ocular radiation exposure [22, 30]. We evaluated the CRR in gastric ESD as a quality indicator in upper GI endoscopy. Inaccurate diagnosis of tumor margins might cause incomplete resection with a positive margin for tumor cells and local recurrence [19, 20, 31]. Therefore, gastric neoplasm demarcation evaluated visually must be very precise. High-resolution endoscopy, magnifying endoscopy, indigocarmine chromoendoscopy, and NBI are used to increase the accuracy of tumor demarcation [31–35]. Therefore, there may be concerns regarding whether the wearing of a face shield affects clarity or the visual field during gastric ESD and thus decreases the accuracy of tumor demarcation and reduces the CRR. In this study, we found that wearing a face shield did not reduce the accuracy of tumor demarcation or delay the procedure time.

This study had some limitations. When a face shield is worn, light may be reflected on the face shield and interfere with the endoscopist’s visual field. In our hospital’s endoscopy room, the lights were turned off, with only the screen of the video processor being turned on, and no direct sunlight. However, while the lighting or brightness of the endoscopy room and the position of the screen may reflect light on the face shield, these effects are likely to differ between endoscopy rooms. In this study, the effects of these differences on the GI quality indicators were not analyzed. Moreover, in this study, the effect of only one type of face shield was investigated. Since various types of face shields are available, their effects on the quality indicators of GI endoscopy may differ. This was a retrospective study of medical records; therefore, it had inherent limitations. In our hospital, endoscopy is required to be performed in strict accordance with guideline recommendations; however, as this was a retrospective study, we could not confirm whether these recommendations were followed or not for some of the patients. However, according to an investigation carried out among our hospital staff, adherence to wearing facial protection was > 95% during the period; therefore, we consider that this limitation did not significantly affect our results. Our results, obtained through examining patient medical records within a short period of time during which facial protection recommendations were strictly followed, are likely to be of value in understanding the effects of changes implemented in response to an unprecedented and globally threatening pandemic.

Experts and medical societies have provided guidelines for the management of COVID-19 infection, and medical workers are required to follow such guidelines to prevent droplet or air transmission. The same management guidelines are also applied when working in the endoscopy room, and the wearing of PPE, including facial protection, is essential for endoscopists [6–10]. However, even when guidelines are strictly followed, endoscopists can experience fear and reluctance to perform endoscopies due to the risk of contracting COVID-19. One study found that gastroenterologists were less fearful of contracting acquired immunodeficiency syndrome (AIDS) approximately six years after the first study about fears of AIDS [36]. This finding indicates that in the unprecedented COVID-19 pandemic era, more research and advances in treatment for COVID-19 are needed to help alleviate endoscopists’ fears. Further studies are also needed to determine whether wearing a face shield is necessary for the prevention of other infections without degrading the quality indicators of colonoscopy, even after the COVID-19 pandemic has ended.

## Conclusions

Quality indicators of GI endoscopy were not affected due to the wearing of face shields during the COVID-19 pandemic. The additional use of face shields to prevent COVID-19 transmission did not reduce the quality of GI endoscopy during the COVID-19 pandemic.

## Data Availability

The datasets used and/or analyzed during the current study are available from the corresponding author on reasonable request.

## Abbreviations

ADRs: Adenoma detection rates
ANDR: Advanced neoplasia detection rate
APC: Adenoma per colonoscopy
COVID-19: Coronavirus disease 2019
CRC: Colorectal cancer
CRR: Complete resection rate
ESD: Endoscopic submucosal dissection
GI: Gastrointestinal
NBI: Narrow band image
PCCRC: Post-colonoscopy colorectal cancer
PDR: Polyp detection rate
PPE: Personal protective equipment
SARS-CoV-2: Severe acute respiratory syndrome coronavirus 2
SSLDR: Sessile serrated lesion detection rate
